# Gene therapy of Dent disease type 1 in newborn ClC-5 null mice for sustained transgene expression and gene therapy effects

**DOI:** 10.1038/s41434-024-00490-w

**Published:** 2024-09-25

**Authors:** Pin Lyu, Manish Kumar Yadav, Kyung Whan Yoo, Cuili Jiang, Qingqi Li, Anthony Atala, Baisong Lu

**Affiliations:** Wake Forest Institute for Regenerative Medicine, Wake Forest University School of Medicine, Winston-Salem, NC USA

**Keywords:** Kidney diseases, Targeted gene repair

## Abstract

Dent disease type 1 is caused by changes in the chloride voltage-gated channel 5 (*CLCN5*) gene on chromosome X, resulting in the lack or dysfunction of chloride channel ClC-5. Individuals affected by Dent disease type 1 show proteinuria and hypercalciuria. Previously we found that lentiviral vector-mediated *hCLCN5* cDNA supplementary therapy in ClC-5 null mice was effective only for three months following gene delivery, and the therapeutic effects disappeared four months after treatment, most likely due to immune responses to the ClC-5 proteins expressed in the treated cells. Here we tried two strategies to reduce possible immune responses: 1) confining the expression of ClC-5 expression to the tubular cells with tubule-specific *Npt2a* and *Sglt2* promoters, and 2) performing gene therapy in newborn mutant mice whose immune system has not fully developed. We found that although *Npt2a* and *Sglt2* promoters successfully drove ClC-5 expression in the kidneys of the mutant mice, the treatment did not ameliorate the phenotypes. However, gene delivery to the kidneys of newborn *Clcn5* mutant mice enabled long-term transgene expression and phenotype improvement. Our data suggest that performing gene therapy on Dent disease affected subjects soon after birth could be a promising strategy to attenuate immune responses in Dent disease type 1 gene therapy.

## Introduction

Dent disease (DD) is an X-linked chronic kidney disorder that mainly affects males. Defects in the kidney proximal tubules cause the disease. Specifically, the dysfunctional kidney proximal tubules cannot reabsorb small molecular weight proteins, water, and calcium filtered into the urine, causing proteinuria, hypercalciuria, nephrocalcinosis, and nephrolithiasis [1]. Dent disease type 1 (DD1, MIM#300009) accounts for about 60% of Dent disease cases and is caused by more than 200 different types of changes in various regions of the *CLCN5* gene (Gene ID: 1184, OMIM #300008) [2], including frameshifts (29.1%), abnormal splicing (12.4%) and premature translational termination (nonsense mutations, 17.5%) [3, 4]. ClC-5, the protein products encoded by human *CLCN5*, can have 746 (NM_000084.5) or 816 (NM_001127898.4) amino acids (the C-terminal 746 AAs of the two isoforms are identical) due to alternative splicing. Both isoforms function as electrogenic Cl-/H+ exchangers and play crucial roles in receptor-mediated endocytosis in proximal tubule epithelial cells [5–9].

To develop a cure for DD1 by targeting the molecular etiology, we recently generated a ClC-5 null mouse model by injecting CRISPR/Cas9 ribonucleoprotein into mouse fertilized eggs, successfully deleting 95% of the mouse *Clcn5* coding region [10]. The mutant mice exhibited evident DD1 phenotypes. We used lentiviral vectors (LV) to deliver human *CLCN5* cDNA into the kidneys of adult mutant mice and found that the expression of the human ClC-5 protein ameliorated all parameters we examined [10]. However, the therapeutic effects disappeared 4 months after LV delivery. A second dose of *CLCN5* LV treatment did not result in ClC-5 expression or therapeutic effects, although delivery of GFP LV to these *CLCN5* LV-treated mice resulted in GFP expression. The results suggested that an immune response most likely developed against the expressed ClC-5 protein.

Dendritic cells (DCs) mediate adaptive immune responses to transgene products [11–15]. DCs are present in the renal tubulointerstitium [16], and could be transduced by LVs to express ClC-5 in these cells. The expression of ClC-5 in DCs could mediate immune responses to exogenous ClC-5. Using tubule-specific promoters to restrict the expression of ClC-5 to tubular cells, rather than using the ubiquitously active EF1 alpha promoter in our previous study, may restrict the exogenous ClC-5 to tubular cells, where ClC-5 expresses and functions [5–9]. The sequences of several proximal tubule-specific promoters, including mouse *Npt2a* [17, 18], mouse *Sglt2* [19, 20], and human *SGLT2* promoter [21, 22], have previously been defined. Whether these promoters can drive ClC-5 expression to improve the phenotypes of ClC-5 null mice and mitigate immune responses is unknown. Additionally, miR-142-3p, which inhibits the expression of mRNAs containing miR-142-3p target sequences in their 3’ untranslated region (3’ UTR), is highly expressed in DCs but not in kidney proximal tubular epithelial cells [23, 24]. It is shown that adding miR-142-3p target sequences to transgenes inhibits transgene expression in DCs and immune responses to transgene products [25–27]. Thus, adding miR-142-3p target sequences to the 3’ UTR of *CLCN5* cDNA is expected to inhibit possible leaky *CLCN5* expression in DCs, yet maintain *CLCN5* expression in kidney tubular cells since they do not express miR-142-3p [23, 24].

The immune system in neonates is functionally compromised [28]. It was found that viral vector-mediated gene delivery in neonatal animals but not in adults resulted in long-term gene expression [29–35]. Since prenatal genetic diagnosis of DD1 is possible [36], performing gene therapy in neonatal DD1 patients may avoid immune responses to transgene products. It is unknown whether performing DD1 gene therapy in neonatal mutant mice will correct the DD1-like phenotype and maintain long-lasting gene therapy effects.

Here we tested two strategies to achieve long-lasting gene therapy effects in DD1 gene therapy. In one strategy, tubule-specific promoters and MiR-142-3p target sequences were used to minimize transgene expression in DC cells and minimize immune responses to the transgene products. In another strategy, gene therapy was performed in neonatal mice to examine whether immune tolerance to the transgene can be induced to achieve long-lasting gene therapy effects.

## Materials and methods

### Animal study

Animal experiments were conducted in accordance with the National Research Council Publication Guide for Care and Use of Laboratory Animals and approved by the Institutional Animal Care and Use Committee of Wake Forest University Health Sciences (Animal protocol numbers A19-053 and A22-043). The ClC-5 null model was generated by our group, and mutant mice used in the study were generated by mating heterozygous females (in C57BL/6NJ background) with FVB/NJ males, as reported previously [10]. Mice housing, genotyping, retrograde ureteral injection, and euthanasia were performed as reported previously [10]. Genotypes were further verified by the presence (in wildtype) or absence (in mutants) of endogenous mouse *Clcn5* mRNA.

### DNA constructs

The LV transfer plasmid for expressing ClC-5 under the EF1 alpha promoter (pCSII-hCLCN5) was described in our previous study [10]. The transfer plasmids for expressing ClC-5 under the three tubule-specific promoters (pCSII-mNpt2a-hCLCN5, pCSII-mSGlt2-hCLCN5, pCSII-hSGLT2-hCLCN5) were generated by replacing the EF1 alpha promoter of pCSII-com-hCLCN5-MiR142T with the respective tubule-specific promoters. The transfer plasmids contained the com aptamer sequence to increase packaging efficiency and target gene expression [37], as well as 4 copies of MiR142-3p target sequences in the 3’ UTR of h*CLCN5* cDNA to inhibit possible transgene expression in DC cells [23, 24]. The promoter sequences for mouse *Npt2a*, mouse *Sglt2*, and human *SGLT2* reported previously [17, 20, 21] were amplified from mouse genomic DNA (for m*Npt2a* and m*Sglt2*) or HEK293T cells (for h*SGLT2*) using CloneAmp HiFi PCR Premix (Takara, Mountain View, USA; catalog # 639298). The PCR products were inserted between the XhoI-XbaI sites of pCSII-com-hCLCN5-MiR142T to replace the EF1 alpha promoter by In-Fusion Cloning (Takara, Mountain View, USA). Construction strategies, primer sequences, and promoter sequences were detailed in Supplementary Tables S1, S2, and S3.

### Lentiviral vector production, purification, quantification, and transduction

Lentiviral transfer plasmid DNA with different promoters was used to produce lentiviral vectors expressing ClC-5, which were generated with the third-generation packaging system as we described previously [10]. Briefly, 12 µg lentiviral transfer plasmid DNA (pCSII-hCLCN5, pCSII-mNpt2a-hCLCN5, pCSII-mSGlt2-hCLCN5, or pCSII-hSGLT2-hCLCN5), 14 µg pMDLg/pRRE, 6 µg pMD2.G, and 4 µg pRSV-Rev were transfected into 13 million HEK293T cells seeded in 15-cm dishes one day before transfection. For transfection, DNA in 1 ml Opti-MEM and 108 µl polyethyleneimine (1 mg/ml, PEI, Synchembio, Cat # SH-35421) in 1 ml Opti-MEM were mixed and incubated at room temperature for 15 min before adding to the cells. Twenty-four hours after transfection, the medium was changed to 15 ml Opti-MEM, and the lentiviral vectors were collected 48 h and 72 h after transfection. The combined supernatants were spun for 10 min at 500 g to remove cellular debris. The vectors in the cleared supernatant were concentrated with the KR2i TFF System (KrosFlo® Research 2i Tangential Flow Filtration System, Spectrum Lab, Cat. No. SYR2-U20) to 3-5 ng/ng p24, as we previously described [10, 38]. The vectors were quantified by p24 (a capsid antigen)-based ELISA (Cell Biolabs, QuickTiter™ Lentivirus Titer Kit Catalog Number VPK-107), aliquoted into 100 µl/tube and frozen at −80 °C for future use as described previously [10]. For lentiviral vector transduction of cultured cells, vectors (equivalent to 10 ng p24 protein) were added to 2.5 ×10^4^ cells grown in 24-well plates in the presence of 8 μg/ml polybrene. The medium was replaced with the normal medium 12 to 24 h following treatment.

To determine the transduction units (TU) of the LV preparations, 2.5 × 10^4^ HEK293T cells seeded 24 h before transduction were transduced by LV of 100 pg p24. 48 h after transduction, genomic DNA was collected and the copy number of codon optimized *CLCN5* DNA (distinguishable from endogenous *CLCN5* sequence) was quantified by droplet digital PCR. PCR primers specific for the codon optimized *CLCN5* (hCLCN5-F and hCLCN5-R, see Supplementary Table S2 for sequences) were used to only detect the vector DNA. Droplet digital PCR was performed with the QX200 Droplet Digital PCR Systems (Bio-Rad Laboratories, Hercules, CA, USA) in 22 μL volume, containing 1 μL template ( < 200 ng/μL), along with ddPCR Supermix for Primers (Bio-Rad Laboratories) at 1X final concentration, 0.25 µM of each primer and nuclease-free water to reach the final volume. Two LV preparations were assayed to be 108 and 267 TU/pg p24 respectively. These values were within the ranges reported in the literature [39].

### Retro-grade ureter injection

Retro-grade ureter injection with adult mice was performed as described previously [10, 40–45]. Briefly, mice anesthetized with 3% isoflurane were made a 2-cm flank incision to expose the left kidney. The ureter below the injection site was clamped with an atraumatic vascular clip (S&T Vascular Clamps Cat# 00400-03, Fine Science Tools, Heidelberg, Germany) to prevent leakage of the injections to the bladder. Lentiviral vectors ( ~ 100 µl/kidney, 2–4 ng p24/µl) were injected into the ureter just below the ureteropelvic junction with a 30-gauge 0.5-inch needle connected to a 1 ml syringe. The clamp was removed 15 min following the injection, followed by the closure of the muscle and the skin in two layers with absorbable 5-0 Vicryl^®^ suture. The procedure was then repeated on the right kidney. Before the mice awakened, 5–10 mg/kg carprofen and buprenorphine SR (0.5–1.0 mg/kg) were delivered via subcutaneous injections. 24 and 48 h after the surgery, 5–10 mg/kg carprofen was injected for pain control. The mice were singly housed to prevent wound damage by cage mates.

### Percutaneous intrarenal injection of newborn mice

The injection was done 1 day after birth as described previously [46]. Both kidneys of all male pups from a litter were injected. The newborn mice were anesthetized by hypothermic anesthesia. To inject the left kidney, firm but gentle pressure was applied to the left hind leg. The left kidney was anatomically located inferior to the spleen on the left lateral side of the pup’s abdomen, which was visible by naked vision upon applied pressure. The needle was inserted into the kidney with the tip facing away from the abdomen (insulin syringe with a 29 G needle, BD Ultra-Fine™). Ten microliters of *CLCN5* lentiviral vectors (3 ng/µl p24) were slowly injected into the left kidney. The procedure was repeated on the right kidney. After injection, the pups were put in a cage on a heating pad and observed for at least 15 min to confirm there was no intra-abdominal bleeding. The injected pups were returned to their home cages and mixed well with the un-injected female pups for 3 min to facilitate the mother to take care of all pups. At last, the mother was kept together with all the pups without disturbance.

### Urine collection

Mice were housed in Hatteras Instruments Model MMC100 Metabolic Mouse Cages (Hatteras Instruments Inc., Cary, NC) for 24 h for urine collection. The urine samples were briefly spun at 1000 g for 5 min to remove possible particles. Urine volume was measured by a 200 µl pipette.

### Urine biochemistry

Urinary calcium concentrations were determined with the Calcium Assay Kits (Colorimetric) (ab102505, AbCam). Urine samples from mutant mice with and without treatment were diluted 20 times with water before the assay. Urinary total protein concentration was determined using Pierce^TM^ BCA Protein Assay kits (Cat#23225). Urine samples from untreated mutant mice were diluted 40 times with water before protein assays; those from treated mutant mice were diluted 20 times. If at 20 times dilution the reads fell outside the ranges of the standards, the samples were diluted 40 times. All measurements were performed according to the manufacturer’s instructions. Urinary Albumin was detected with the ELISA kit from AbCam (ab108792). The urine samples were diluted 2000-fold with sample buffer for analysis. ELISA was performed following the instruction of the manufacturer.

### SDS-PAGE and western blotting analyses

Each kidney was first cut into two longitudinal parts along the middle plate, and then each part was divided into 6 pieces of similar mass by slicing in the direction perpendicular to the original cut. Tissue pieces were stored at −80 °C for DNA, RNA, and protein extraction. Kidney tissues were lysed in RIPA buffer with protease inhibitors (0.5 mm PMSF and 1x Complete Protease Inhibitor Cocktail, Roche Diagnostics Corporation, Indianapolis, IN, USA), and phosphatase inhibitors (50 mM NaF, 1.5 mM Na3VO_3_), and the lysates were mixed with Laemmli buffer for SDS-PAGE for Western blotting analyses. Urine samples were lysed directly in 2x Laemmli buffer (1:1 in volume) containing protease inhibitors and phosphatase inhibitors. To detect ClC-5, two antibodies were used. For experiments testing tubule-specific promoters, ClC-5 Rabbit polyclonal antibody from GeneTex (GTX53963, 1:500, Irvine, CA) was used. For the rest experiments, ClC-5 Rabbit polyclonal antibody from Proteintech (26812-1-AP, 1:1000, Rosemont, IL) was used. Anti-β-actin antibody was from Sigma (A5441, 1:5000; St Louis, MO), CC16 rabbit polyclonal antibody from BioVendor (RD181022220-01, 1:500, Asheville, NC), albumin goat polyclonal antibody from Bethyl Laboratories (A80-129A,1:1000, Montgomery, TX), vitamin D binding protein (DBP) Rabbit polyclonal antibody from Proteintech (16922-1-AP, 1:1000, Rosemont, IL), megalin rabbit polyclonal antibody from Abcam (ab76969, 1:1000, Boston, MA). HRP conjugated anti-mouse IgG (H + L) (Thermo Fisher Scientific, Cat No. 31430, 1:5000, Waltham, MA) and anti-rabbit IgG (H + L) (Cat No. 31460, 1:5000) secondary antibodies were used in Western blotting. Chemiluminescence reagents (Thermo Fisher Scientific) were used to visualize the protein signals with an iBright1500 (Thermo Fisher Scientific). Densitometry was performed with ImageJ (1.54d, NIH).

### Immunofluorescence analyses

Kidney tissues were fixed in 4% paraformaldehyde/PBS at 4 °C overnight, dehydrated, and embedded in paraffin. Paraffin-mounted sections of 5–8 μm were prepared for histologic and immunofluorescence analyses. For immunofluorescence staining, deparaffinized and rehydrated sections were incubated with ClC-5 primary antibodies (1:200) following blocking and were then incubated with CF-594 conjugated secondary antibody (Biotium, Fremont, CA). Sections were mounted in a mounting medium with DAPI (Vector Laboratories, Burlingame, CA). Images were acquired with an Axio M1 microscope equipped with an AxioCam MRc digital camera (Carl Zeiss, Thornwood, NY). Different images were assembled into one file with Adobe Photoshop, with subsequent resizing, rotation, and cropping. The percentages of ClC-5 positive areas were quantified using ImageJ (1.54d). Square boxes, centered on the glomeruli and approximately nine times the area of the glomeruli, were drawn to define the total area for analysis. ClC-5 positive areas within these boxes were outlined using freehand selections. Both the areas of the boxes and the freehand-selected ClC-5 positive areas were measured with ImageJ. The percentage of ClC-5 positive areas relative to the square box areas was then calculated.

### Vector DNA detection

A piece of kidney tissue was used for genomic DNA isolation using a DNeasy Blood & Tissue Kit (Qiagen, Germantown, MD). To detect lentiviral vector DNA, the Psi or codon-optimized *hCLCN5* sequence from the lentiviral vector was detected by qPCR, using custom synthesized Taqman probes (Thermo Fisher Scientific). Mouse *Gapdh* Taqman probe was used as an internal control for gDNA PCR. TaqMan Universal PCR Master Mix (Thermo Fisher Scientific) was used for qPCR detection with a QuantStudio3^TM^ or ABI 7500 instrument.

### RNA isolation and RT-qPCR analyses

A RNeasy Plus Mini Kit (Cat No. 217004, QIAGEN) was used to isolate total RNA from tissues and cultured cells. The QuantiTect Reverse Transcription Kit (QIAGEN) was used to reverse-transcribe the RNA to cDNA. Mouse *Ppib* (primers listed in Table S2) was used as the reference gene in semi-quantitative PCR. *mGapdh*, *mClcn5*, and codon-optimized *hCLCN5* Taqman probes were used for qPCR.

### Statistical analysis

Statistical assessments were performed on urinary parameters using GraphPad Prism (V10) software. Data are presented as median ± standard deviation (SD). For analyses comparing two groups, Mann-Whitney tests were performed. The significance was set at *p < 0.05, **p < 0.01 and ***p < 0.001.

## Results

### Tubule-specific promoters were unable to ameliorate DD1 phenotypes in ClC-5 null mice

**Fig. 1:**
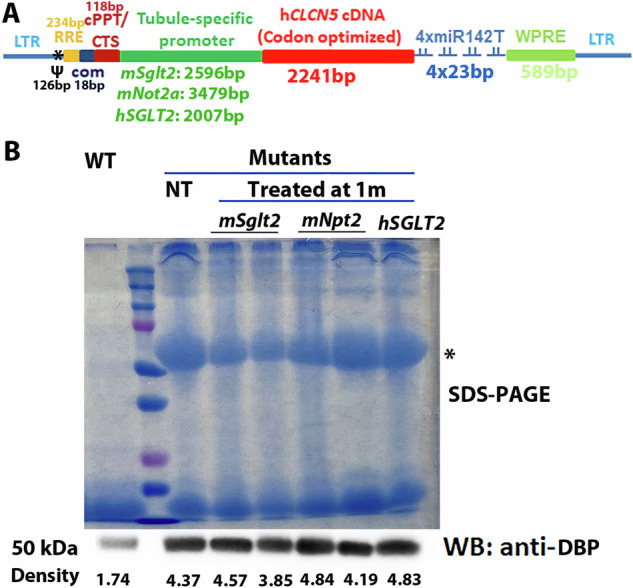
Using tubule-specific promoters for gene therapy in ClC-5 mutant mice. **A** Configuration of the lentiviral vector DNA. Four copies of miR-142 target sites were included in the 3’ UTR of *CLCN5* cDNA to avoid transgene expression in dendritic cells. **B** SDS-PAGE and Western blotting analyses of urinary proteins after treatment with LVs containing tubule-specific promoters. The integrated intensity of each Western blotting band was listed. Western blotting analysis was not repeated. WT wild type, NT not treated, WB Western blotting, DBP vitamin D binding protein.

h*CLCN5* LVs, whose h*CLCN5* expression was controlled by mouse *Npt2a* [17, 18], mouse *Sglt2* [19, 20], and human *SGLT2* promoter [21] respectively (Fig. 1A), were prepared for experiments. 500 ng p24 tubule-specific h*CLCN5* LVs were delivered to both kidneys of mutant mice (two mice per promoter) via retro-ureter injection, as we did previously [10]. One month after the treatment, urine was collected from the treated mice. SDS-PAGE analysis of the urine proteins revealed that none of the treated mice showed an evident reduction in the intensity of the ~60 kDa band, which was found to be a good indication of phenotype improvement (Fig. 1B, top) [10]. Consistent with the SDS-PAGE data, Western blotting analysis of DBP in the urine failed to observe a reduction of the protein in treated mice (Fig. 1B, bottom, see Fig. S1 for uncropped Western blotting image). These observations were in sharp contrast to our previous study, where 100% of the 21 mutants treated with EF1 alpha promoter-controlled *CLCN5* LV showed an evident reduction of urinary proteins.

**Fig. 2:**
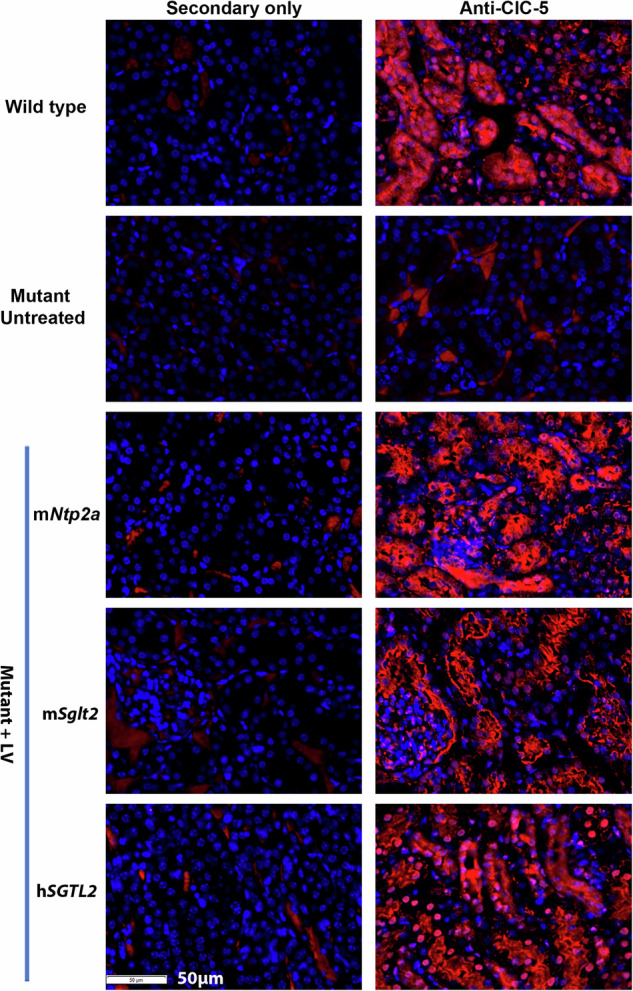
Detection of ClC-5 protein expression following delivery of *CLCN5* LV controlled by tubule-specific promoters. ClC-5 signals were shown in red. Nuclei were stained by 4’, 6-diamidino-2-phenylindole (DAPI, shown in blue).

To check whether the lack of effects was the result of delivery failure, we euthanized the mice and checked the expression of ClC-5 protein by immunostaining. ClC-5 was not detected in kidneys from mutant mice without treatment but was detected in wildtype mice and mutant mice treated with the tubule-specific LVs (Fig. 2). The lack of phenotype improvement despite successful ClC-5 protein expression in the treated kidneys suggested that these promoters might not be suitable for driving *CLCN5* expression for DD1 gene therapy. Thus, this strategy was not pursued further. The *CLCN5* promoter would be the best choice for our purpose. However, the regulatory elements necessary for controlling the tubule-specific expression of *CLCN5* have not been fully defined. Since the tubule-specific promoters did not produce observable therapeutic effects, we decided not to further examine the effects of MiR142-3p target sites in the study. The observation of ClC-5 expression in the LV-treated mice is consistent with the observation that MiR142-3p is specifically expressed in DC cells [23, 24].

### Gene therapy in newborn mutant mice achieved sustained therapeutic effects

**Fig. 3:**
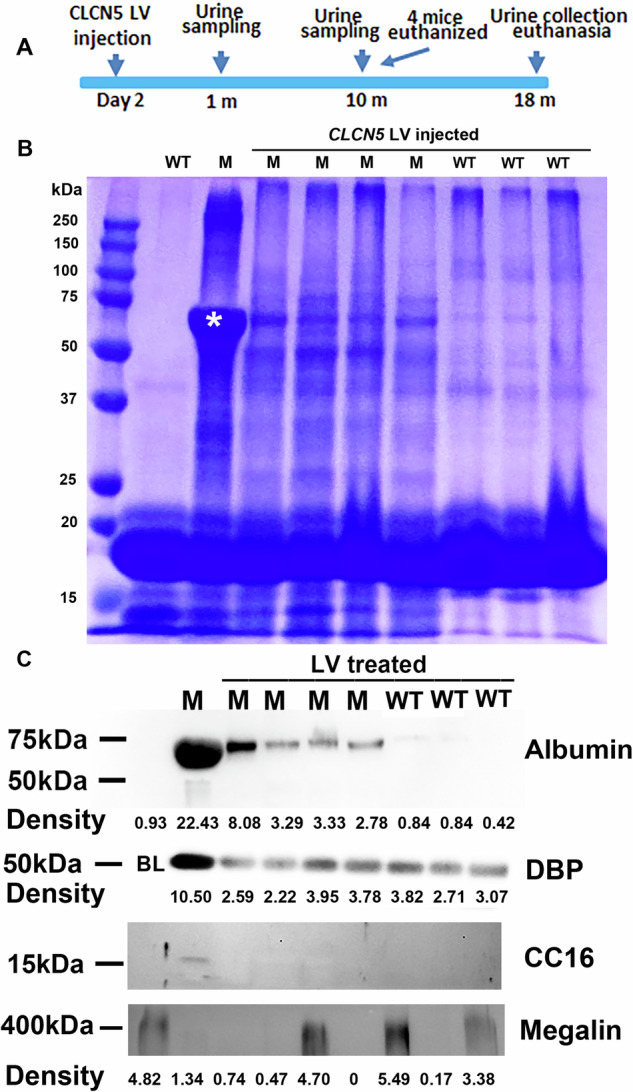
Urine protein analyses 1 month following gene delivery to newborn ClC-5 null mice. **A** Experiment scheme. **B** SDS-PAGE analysis of urinary proteins one month following gene delivery. **C** Western blotting analyses of urinary proteins one month following gene delivery. In DBP WB analysis, wild-type urine was not loaded and indicated by BL (blank). For **A**, **B**, equal urine volumes were analyzed for each sample. The images represent at least two repeats. M mutant, WT wild type, DBP Vitamin D binding protein, CC16 Club cell secretory protein. The integrated intensity of each Western blotting band was listed except for CC16, which only showed a band in the untreated mutant mouse.

We then tested whether gene therapy in newborn mice can achieve long-lasting effects using a scheme depicted in Fig. 3A. Since the h*CLCN5* LV with the EF1 alpha promoter corrected the phenotypes in adult mice [10], it was used in newborn DD1 gene therapy. h*CLCN5* LV (30 ng p24/kidney) was delivered to both kidneys of neonatal mice 1 day after birth, by percutaneous intrarenal injection [46]. To examine whether the injection successfully delivered LV vectors to the kidney, we euthanized one mouse at the age of 4.5 months and checked the presence of vector DNA sequences (two regions, the psi sequence, and the human *CLCN5* cDNA sequence) in the kidney, liver, spleen, testis, heart, brain and the lung. We detected both sequences in the kidney but not the other organs (Table 1). The cycle threshold number for the LV-injected kidney was 10 cycles less than those of the other organs from the same mouse, which was similar to the Ct number of the kidney without LV injection. The data confirmed that the vectors were successfully injected into the kidney and the vector load of the other organs was near or below the detection limit of our qPCR assay. If there was any in the other organs, the vector load was lower than 1/3000 of the kidney level (a difference of 10 cycles).

**Table 1:** Ct numbers for organs with and without LV injection.

	Kidney (No LV)	LV injected						
		Kidney	Brain	Heart	Liver	Lung	Spleen	Testis
*GAPDH*	14.2	14.8	16.3	16.6	15.6	15.1	14.4	14.7
*hCLCN5*	35.5	25.0	35.2	35.9	36.1	36.1	36.0	35.8
*Psi*	34.7	23.8	33.4	34.0	33.5	33.9	34.5	34.1

Four mutant mice and three wild-type mice treated by the *CLCN5* LV were housed for continued observations. At the age of 1 month, urine was collected, and urine proteins were analyzed by SDS-PAGE and Western blotting. In SDS-PAGE, the strong 60 kDa band observed in untreated mutant mice (marked by an asterisk) was greatly reduced in all 4 treated mutant mice (Fig. 3B), although the band was still stronger than in the treated wild-type mice. Western blotting confirmed that gene therapy in mutant mice reduced urinary albumin, vitamin D binding protein B (DBP), and CC16 (Fig. 3C, see Supplementary Fig. S2 for uncropped Western blotting images), proteins known to be increased in the urine of Dent’s patients and *Clcn5* knockout mice. We also detected megalin in the urine samples as a loading indication. Consistent with the observation that megalin decreased with ClC-5 deficiency, megalin was detected in wild-type mice but not in untreated mutant mice. Nevertheless, megalin was detected in samples where albumin, DBP, and CC16 were undetectable, confirming sample loading in these samples.

**Fig. 4:**
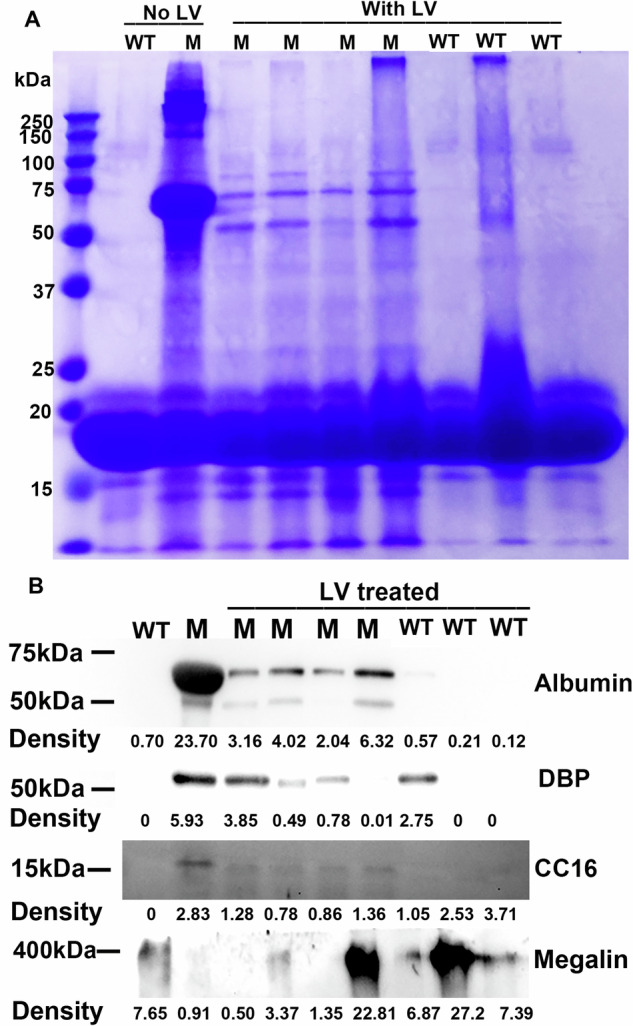
Analyzing urine proteins 10 months following gene delivery. **A** SDS-PAGE analysis of urinary proteins. **B** Western blotting analyses of urinary proteins. The integrated intensity of each Western blotting band was listed. The images represent data from two experiments. For (**A**, **B**), Equal urine volumes were analyzed for each sample. M mutant, WT wild type, DBP Vitamin D binding protein, CC16 Club cell secretory protein.

Similar analyses were performed at the age of 10 months. Urine proteins of treated mutants were still reduced compared with those of urine from untreated mutants (Fig. 4, see supplementary Fig. S3 for uncropped Western blotting images). We compared the urine proteins of treated mutant mice 10 and 18 months following gene delivery using SDS-PAGE, and observed no evident changes in intensities of the band around 60 kDa between 10 and 18 months following gene delivery (Supplementary Fig. S4). It seemed that the gene therapy effects remained for 18 months following gene delivery, contrary to gene delivery to adult mice where gene therapy effects diminished 4 months following gene delivery [10].

Attempts to compare total urinary protein (using the BCA method) and urinary calcium excretion were difficult due to several factors. First, it was difficult to obtain an accurate estimate of diuresis in a study lasting over 18 months, especially on aged mice. Second, the urine samples precipitate during long-term storage, causing the loss of soluble proteins and calcium as previously observed [47]. Accordingly, we observed large intra-group variation in urine samples of untreated mutant mice that had been stored for a long time, making it difficult to observe any changes in urinary protein or calcium in treated mutant mice. We compared urinary albumin protein (ELISA) and calcium concentrations between treated mutants and wildtype mice in samples collected 10 and 18 months following gene delivery since these samples were collected at similar time points and preserved under similar conditions. We found that urine albumin and calcium concentrations of mutant mice were both higher than those of wildtype mice (Supplementary Fig. S5), consistent with our SDS-PAGE and Western blotting results shown in Figs. 3 and 4. The data suggest that the treatment did not completely restore the proximal tubule functions in mutant mice.

### Transgene expression was preserved in all treated mice throughout the study

To corroborate our observation of gene therapy effects, we assayed LV vector and ClC-5 expression in h*CLCN5* LV-treated mice, taking advantage of the fact that the exogenous LV-derived *CLCN5* cDNA was codon optimized to be distinguishable from the endogenous human *CLCN5* and mouse *Clcn5* sequences. LV genome was detected in LV-treated mice at 10 months (2 mutant and 2 wildtype mice) and 18 months (2 mutant and 1 wildtype mice) after treatment. In qPCR analyses, the cycle threshold numbers for the codon-optimized *hCLCN5* cDNA sequence (from the delivered LV) from untreated and treated mice were ≥34 and ≤27 respectively, indicating the presence of LV genomic DNA in all treated mice.

**Fig. 5:**
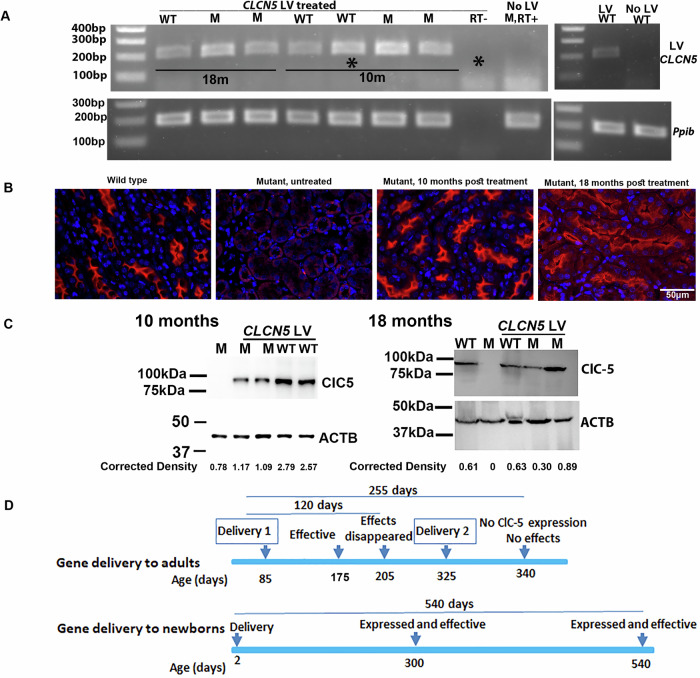
Long-term expression of ClC-5 in the kidneys of LV-treated mutant mice. **A** RT-PCR analysis of kidney mRNA. An expected band of 209 bp could only be detected in mice with LV injection but not in mice without LV injection. M mutant, WT wild type, RT- reverse transcriptase negative, RT+ reverse transcriptase positive. mRNA of lanes with an * was from the same mouse. The number of months following LV injection (10 m and 18 m) was indicated. *Ppib* was used as an internal control. **B** Immunofluorescent analysis of ClC-5 protein in the treated kidneys. ClC-5 signals were shown in red. Nuclei were stained by 4’, 6-diamidino-2- phenylindole (DAPI, shown in blue). **C** Western blotting analysis of ClC-5 protein in kidney tissues 10 and 18 months after gene therapy. Beta Actin (ACTB) was used as a loading control. Normalize intensity was the ratio of the ClC-5 intensity versus ACTB intensity. Western blotting experiments were repeated once. **D** Comparison of gene delivery in adult and newborn mutant mice.

We then examined exogenous h*CLCN5* cDNA expression in the kidney. A PCR band with the expected size could be amplified from cDNAs of all 7 LV-treated mice (4 mutant and 3 wildtype mice), but not from untreated wild-type or mutant mice, or mRNA from LV-treated mice without reverse transcription (RT-) (Fig. 5A). The data showed that 18 months following gene delivery, the transgene was still transcribed, regardless of whether mutants or wildtype mice were delivered.

We then examined kidney ClC-5 protein expression by immunofluorescent analyses. ClC-5 was not detected in mutant mice without LV delivery but was detected in all mutant mice with LV delivery 10 and 18 months following *CLCN5* LV delivery (Fig. 5B, see supplementary Fig. S6 for large areas). We also did ClC-5 immunofluorescent staining on kidney sections from wildtype mice with and without LV injection, LV injection did not increase ClC-5 expression in wildtype mice, suggesting post-transcription regulation of ClC-5 expression. We quantified the percentages of ClC-5 positive areas in sections from untreated wild-type mice, untreated mutant mice, and LV-treated mutant mice. The percentages were 6.2%, 0%, and 8.1%, respectively (two sections from two mice in each group were quantified). LV-injected mutant mice showed similar or even slightly higher ClC-5 positive areas compared to wild-type mice, possibly because endogenous ClC-5 in wild-type mice has restricted expression, whereas in LV-injected mice, exogenous ClC-5 expression was ubiquitous due to the EF1 alpha promoter used to drive ClC-5 expression. Consistent with the immunofluorescent results, Western blotting also showed the expression of ClC-5 expression in LV-delivered mutant mice 10 and 18 months after gene delivery (Fig. 5C, see supplementary Fig. S7 for uncropped Western blotting images). 10 months following gene delivery, we observed more ClC-5 protein in LV-treated wildtype mice than in mutant mice. The significance of this observation is unclear due to the limited animal numbers observed. Note that in LV-delivered wildtype mice, we were unaware of how much ClC-5 protein was expressed from the LV-delivered cDNA since the antibody could not distinguish between the endogenous and exogenous ClC-5 proteins. However, the detection of LV-specific *hCLCN5* cDNA in LV-delivered wildtype mice showed that LV-delivered *hCLCN5* was expressed in wildtype mice throughout the study. Expression of ClC-5 protein in gene-delivered mutant mice throughout the study explained the decrease in urinary proteins abundant in mutant mice.

## Discussion

Consistent with observations of immune responses to transgene products in animal studies [11, 25, 27, 29] and in gene therapy clinical trials for α-1-antitrypsin deficiency [48] and Duchenne’s muscular dystrophy [49], we recently found that ClC-5-expressing lentiviral vectors delivered to the kidneys of adult mice only showed therapeutic effects in the first three months [10]. Re-dosing of the same vector failed to mediate ClC-5 expression, contrary to the observations of GFP expression when GFP-expressing vectors were delivered to mice originally receiving *hCLCN5* LV. These observations suggest that immune responses to the transgene product ClC-5 caused the loss of effects 4 months after the gene delivery. In this study, we tested *CLCN5* LV delivery to newborn mice and observed transgene expression and treatment effects during the whole period of the study. Although 18 months was the latest time point examined, we reason that transgene expression and treatment effects should be expected at later ages. This is in sharp contrast to gene delivery in adult mice [10], where treatment effects disappeared 4 months after gene delivery. In that study [10], the mice were euthanized 255 days after the first gene delivery and 15 days after the second gene delivery (Fig. 5D), and no ClC-5 expression could be detected. Considering the loss of gene therapy effects 4 months after gene delivery, loss of ClC-5 expression most likely happened around that time, earlier than mice euthanasia. The two studies used the same mutant strain and lentiviral vector, whereas gene delivery to newborn mice resulted in long-term transgene expression (Fig. 5D), an observation consistent with the long-term reduction of urinary albumin, DBP, and CC16 in treated mice. Although we could not observe a reduction in urinary calcium after gene therapy compared to untreated mutant mice, we postulate that this was most likely the result of urine forming precipitation following long-term storage at -80 degrees. Previously it was observed that calcium and protein were the major components in the precipitates [47]. The reduction of albumin, DBP, and CC16 in Western blotting analyses showed that the treatment was effective. In this study, the transgene was expressed in 100% of treated mice (3 wildtype and 4 mutant mice) throughout the study, suggesting that the cells receiving the gene were not lost with time. The long-term transgene expression suggests that tolerance to transgene products is induced following gene delivery to newborn mice, consistent with the induction of tolerance following gene delivery to newborn animals in other studies [29–35].

Prenatal and neonatal lentiviral vector gene delivery had been performed in mice with sustained high-level transgene expression [50, 51]. One of the mechanisms for the long-term expression could be the reduced immune responses, the results of gene delivery before the maturation of the mouse immune system. It was also found that cell division facilitated lentiviral vector transduction [52]. This observation suggested another benefit of our performing gene therapy in neonatal mice—enhanced delivery efficiency of the lentiviral vectors to the proliferating tubule cells. The safety of the integrating LVs must be considered, especially when delivered to prenatal and neonatal subjects. HIV-based LV (the vector used in this study) did not cause tumors delivered to prenatal or neonatal mice, although EIAV-based LV vectors did in the same study [53]. With the EIAV-based LV vectors, tumors were only observed in the liver but not in the other organs, although systemic delivery was performed.

The tubule epithelium has a low turnover rate [54]. The tubule epithelium is believed to replace aged cells via dedifferentiation and division of the dedifferentiated cells [55, 56]. Since the LV genome integrates into the genome of the tubule epithelial cells, the dedifferentiation and proliferation process will maintain the percentage of cells with LV integration during aging. This explains why ClC-5 can be expressed for the long term from the LV vectors.

We observed that although tubule-specific promoters, including m*Npt2a*, m*Sglt2*, and h*SGLT2*, mediated ClC-5 expression in the tubules of the mutant mice, they failed to improve the DD1 phenotypes. One explanation is that there were subtle improvements but missed our detection. Another possibility is that although these promoters enabled ClC-5 expression in the proximal tubules, these cells were not the authentic ClC-5-expressing tubular cells, and they lacked necessary ClC-5 interacting partners, such as megalin [57] and KIF3B [58], to maintain proper ClC-5 subcellular localization and function. Although the EF1 alpha is ubiquitously active, ClC-5 can be expressed in authentic ClC-5-expressing cells, which explains why *CLCN5* LV with the EF1 alpha promoter worked in our hands. In this experiment, we included target sequences for MiR142-3p in the 3’ UTR of *CLCN5* cDNA. Since the promoters failed to generate detectable therapeutic effects, we did not pursue the effects of including the MiR142-3p target sequences in the constructs. However, ClC-5 was expressed from the vectors with the MiR142-3p target sequences. The data confirmed that the kidney cells do not express MiR142-3p.

Given the absence of a cure for Dent disease at present, this study holds clinical significance. Our previous study suggested that gene therapy in adult Dent patients may induce immune responses to the ClC-5 protein, while this study suggests that gene therapy in babies affected by Dent disease may provide long-lasting effects. Although 18 months post-gene delivery was the latest time point examined, the expression of the transgene at this time made us expect that the gene most likely would be expressed beyond 18 months if the experiment had not been terminated. The age of 18 months in mice is equivalent to about 50 years in humans. If the data can be translated into humans, then performing gene therapy on babies affected by Dent disease would produce long-lasting effects. Since Dent disease is X-linked and prenatal diagnosis is possible [36], administrating the *CLCN5* LV vectors to newborn babies is a feasible choice. Retrograde ureter delivery to the human kidney during ureteroscopy is a minimally invasive procedure and has been performed with a high success rate in babies aged as young as 8 months [59]. More work is needed to determine whether it is possible to perform this procedure on newborn babies. In addition, many other genetic diseases affect the kidney tubules and currently have no cure. Our study may promote the development of treatments for other tubulopathies. If gene therapy for Dent disease-affected babies is successful, the same strategy can be used to treat other tubulopathies.

Our study has limitations. First, the number of animals treated was small. Four mutant mice and three wild-type mice were included in the study. Nevertheless, expression of the transgene 18 months after gene delivery in 100% of the treated mice suggests that the likelihood of losing transgene expression earlier than 18 months is low even if more animals were tested. Another limitation of the study is the inability to detect calcium reduction in the treated mice. This was most likely caused by the formation of precipitates during the long-term storage of the urine samples from untreated mice that we used for comparison.

In summary, this study found that lentiviral vector-mediated *CLCN5* gene delivery to newborn DD1 mice resulted in long-term transgene expression and reduced urinary proteins typically increased in DD1 patients. The study showed that gene therapy in neonates is a promising strategy to minimize immune responses to transgene products.

## Supplementary information

Supplementary data

Supplementary table

## Data Availability

Constructs and primer information are provided in supplementary files. Plasmids are available upon request.
