# Supplementary material for: Gene therapy of Dent disease type 1 in newborn ClC-5 null mice for sustained transgene expression and gene therapy effects
Source: Gene Ther. 2024 Sep 25;31(11-12):563–71. doi: 10.1038/s41434-024-00490-w (PMC11576508; doi:10.1038/s41434-024-00490-w)

**Supplementary data**

**Figure S1**. Uncropped western blotting image for **Fig.1B**. The membrane image with the marker bands was merged with the luminescent image to indicate the size of the protein.


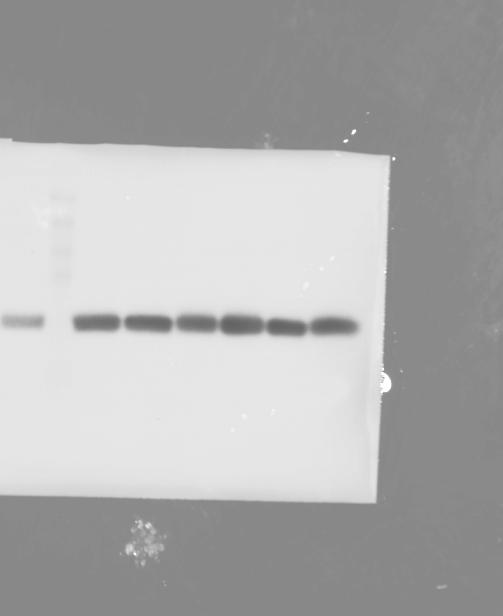


**Figure S2**. Uncropped western blotting images for **Fig.3C**. The membrane image with the marker bands was merged with the luminescent image to indicate the size of the protein.


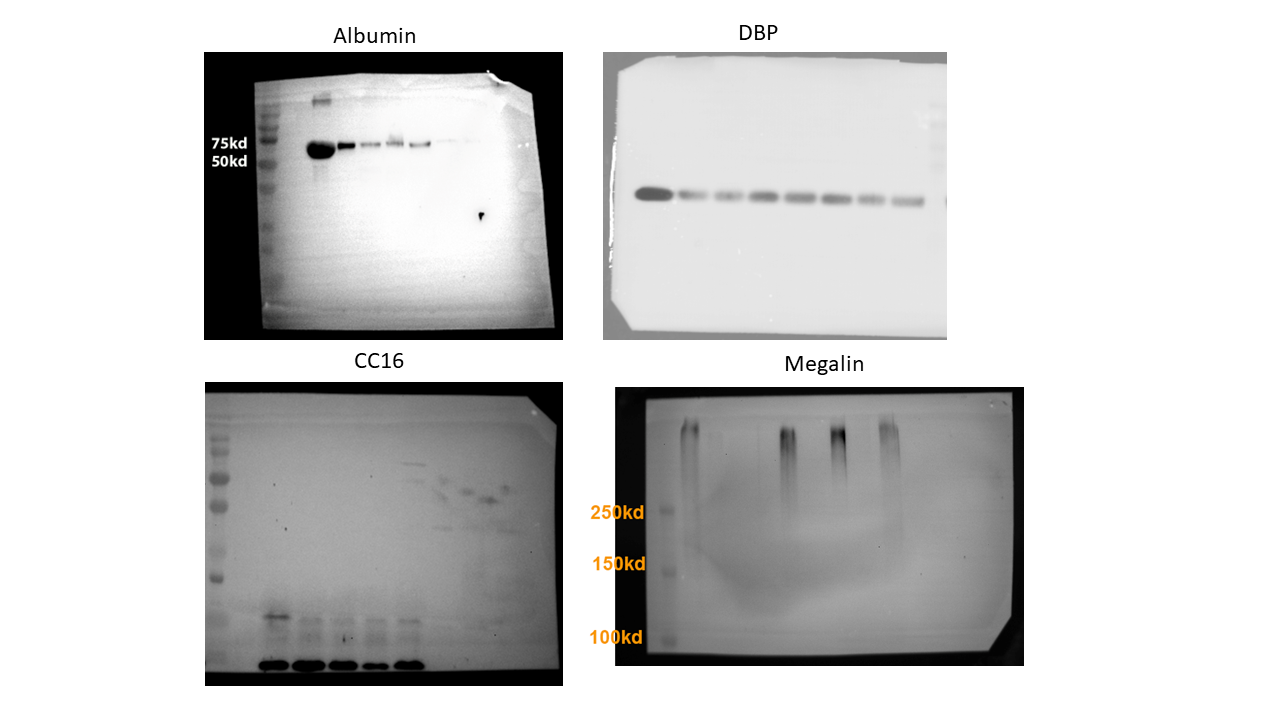


**Figure S3**. Uncropped western blotting images for **Fig.4C**. The membrane image with the marker bands was merged with the luminescent image to indicate the size of the protein.


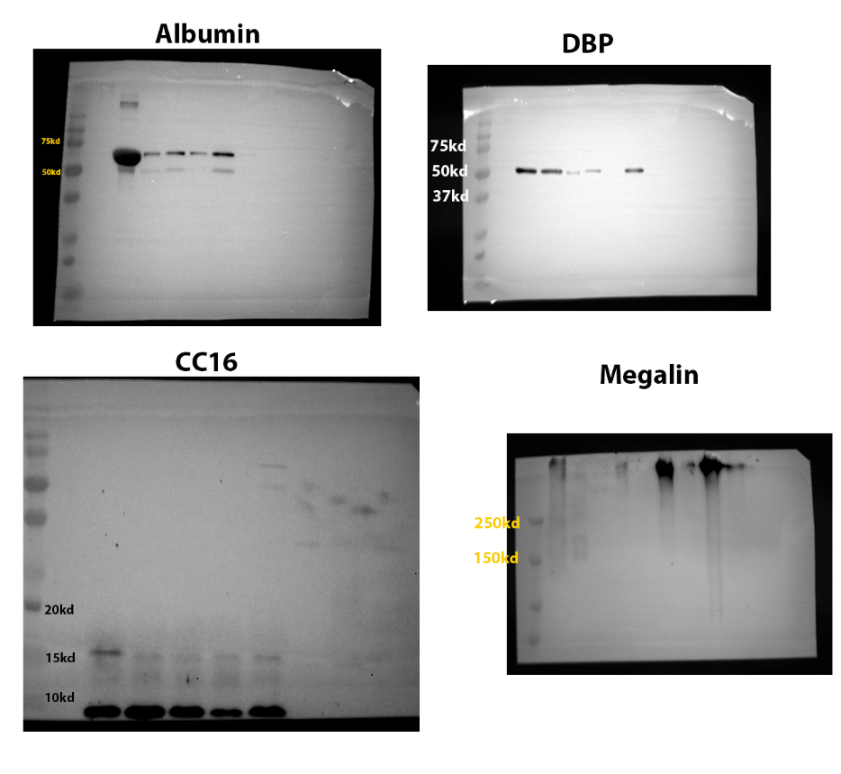


**Figure S4**. SDS-PAGE analysis of urine samples 10 and 18 months after gene delivery from CLCN5 LV treated mutant mice. M: *Clcn5* mutant mice. M1 and M2: two treated mutant mice euthanized 18 months after gene delivery. WT: wild type mouse. Molecular weights of marker proteins were listed on the left.

**
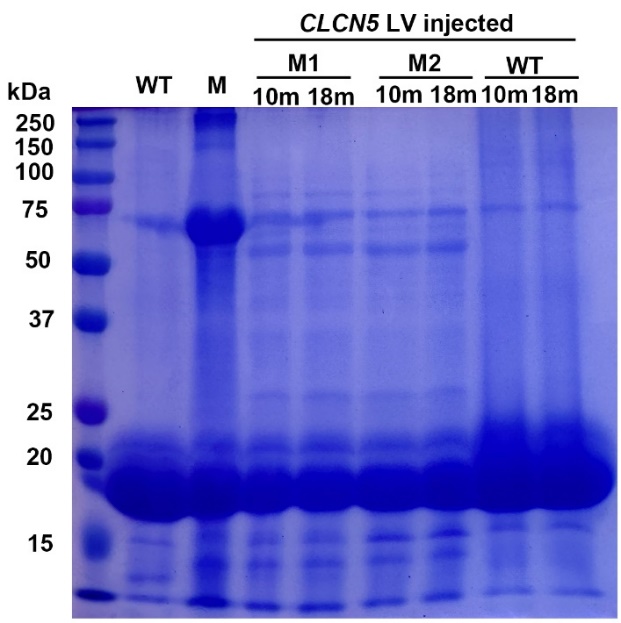
**

**Figure S5**. **Comparison of urinary protein and urinary calcium from mutant and wildtype mice.** Urine samples collected 18 months after gene delivery were indicated by number 18 in parentheses. The other samples were collected 10 months after gene delivery. *: p<0.05 in Mann-Whitney test. Under the dilution factors used, the urinary albumin of wild type mice near the detection limitation, thus the concentration might not be accurate for these samples yet the relationship between mutants and wildtype samples was valid.


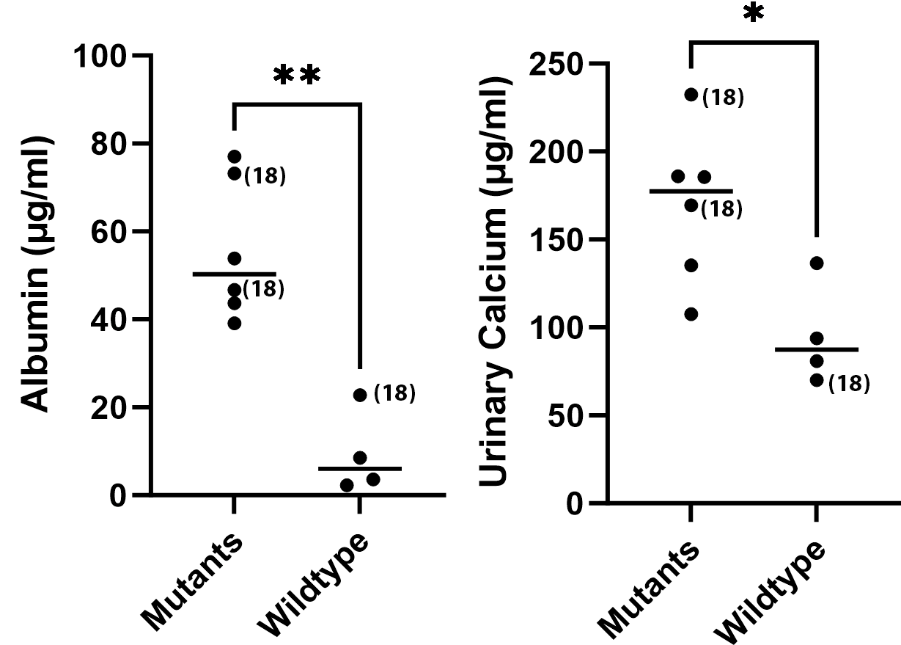


**Figure S6**. Images of large area showing the expression of ClC-5. Scale bar: 200 µm.


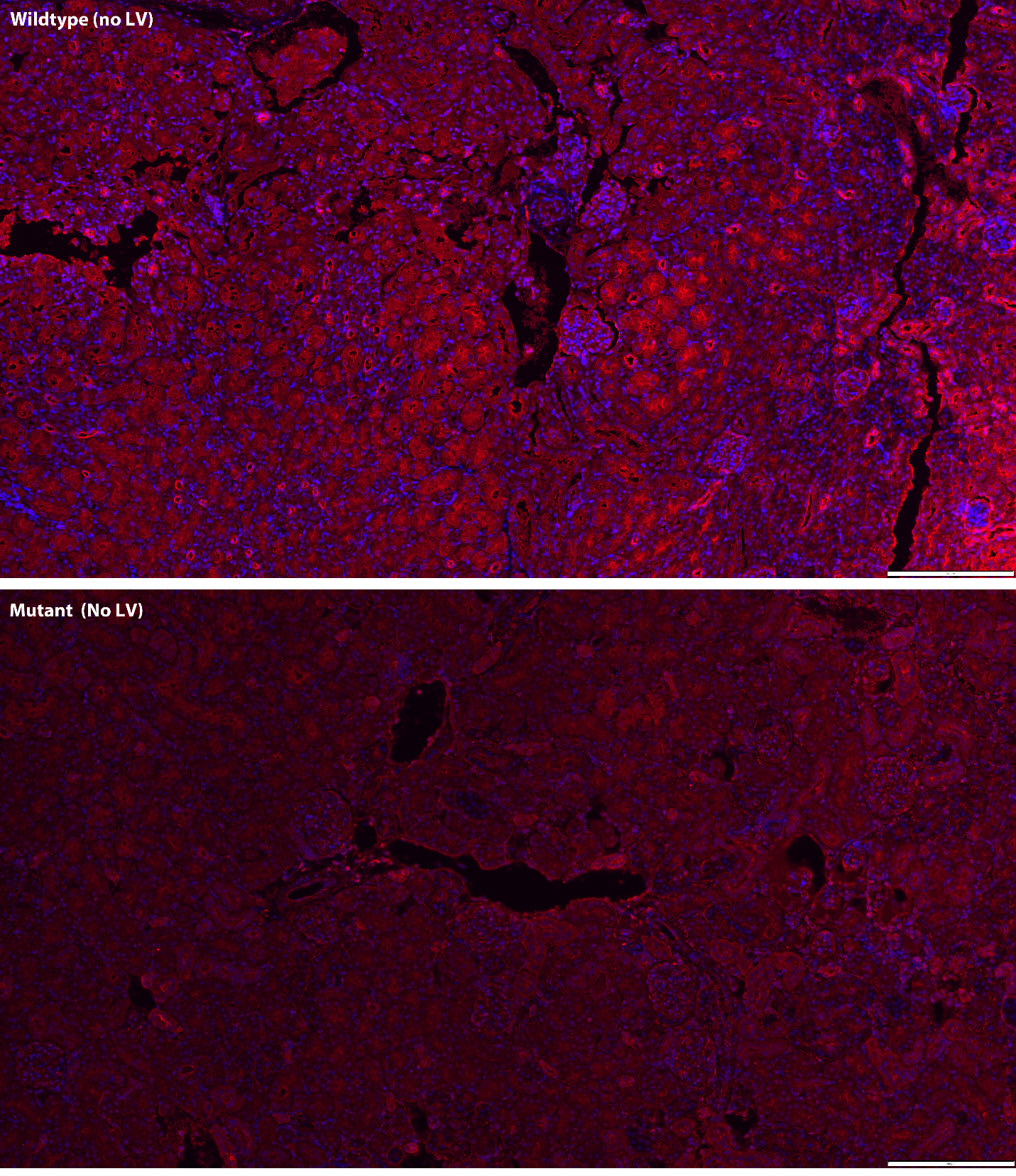


**
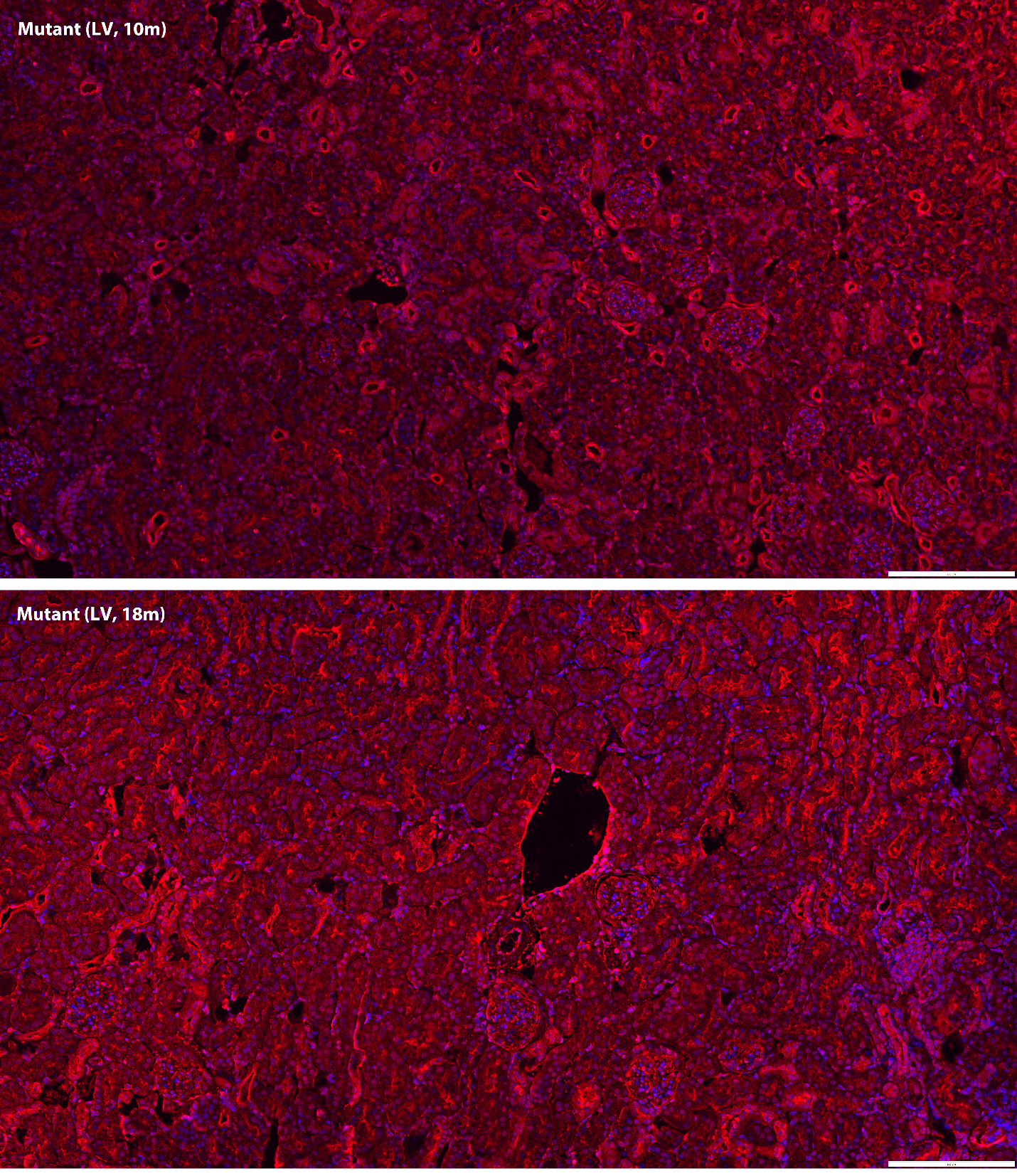
**

**Figure S7**. Uncropped western blotting images for **Fig.5C**. The membrane image with the marker bands was merged with the luminescent image to indicate the size of the protein.


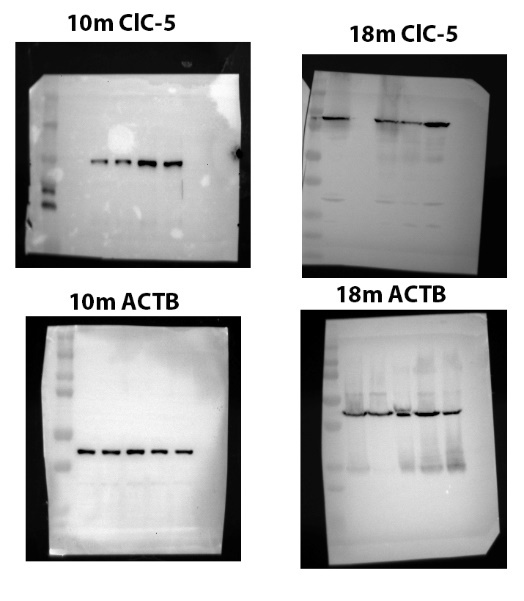

Supplement: Supplementary file 1 — Supplementary data [file 41434_2024_490_MOESM1_ESM.docx]
