# Supplementary material for: Gene therapy of Dent disease type 1 in newborn ClC-5 null mice for sustained transgene expression and gene therapy effects
Source: Gene Ther. 2024 Sep 25;31(11-12):563–71. doi: 10.1038/s41434-024-00490-w (PMC11576508; doi:10.1038/s41434-024-00490-w)
Supplement: Supplementary file 2 — Supplementary table [file 41434_2024_490_MOESM2_ESM.docx]

**Table S1. Construction of LV transfer plasmids with tubule-specific promoters**

| Plasmid name | Promoter Sequence | Construction strategy |
| --- | --- | --- |
| pCSII-com-hCLCN5 | EF1 alpha promoter. | Com for LV-F1 Oligo and Com for LV-R1 oligo were annealed and inserted into the KflI site of pCSII-hCLCN5 by IN-Fusion cloning. |
| pCSII-com-hCLCN5-Mir142T | EF1 alpha promoter. | Mir142-F1 and MIR142-R1 were annealed and inserted between the XbaI and HpaI sites of pCSII-com-hCLCN5. |
| pCSII-mNpt2a-hCLCN5 | Mouse Npt2A promoter. | mNPT2A-F3 and mNPT2A-MR were used as primers to amplify a 1308 bp DNA from mouse genomic DNA, mNPT2a-MF and mNPT2A-R2 were used as primers to amplify a 2220 bp DNA from mouse genomic DNA, the two DNA fragments were linked by overlap extension PCR. The PCR product was then inserted between the two AgeI sites of pCSII-com-hCLCN5-Mir142T by IN-Fusion cloning. |
| pCSII-mSglt2-hCLCN5 | Mouse Sglt2 promoter | mCLCN5-F5 and CLCN5-MR2 were used as primers to amplify a 1943 bp DNA from mouse genomic DNA, CLCN5-MF2 and mCLCN5-R5 were used as primers to amplify a 706 bp DNA from mouse genomic DNA, the two fragments were inserted between the two AgeI sites of pCSII-com-hCLCN5-Mir142T by IN-Fusion cloning. |
| pCSII-hSGLT2-hCLCN5 | Human SGLT2 promoter | hCLCN5-F5 and hCLCN5-R5 were used as primers to amplify a 2039 bp DNA from HEK293T genomic DNA, and was inserted between the two AgeI sites of pCSII-com-hCLCN5-Mir142T by IN-Fusion cloning. |

**Table S2. Primer sequences**

| Primer name | Sequence | Purpose |
| --- | --- | --- |
| Com for LV-F1 | caaccccgaggggacCTGAATGCCTGCGAGCATCCCACgacccgacaggcccg | Annealed to insert into the KflI site of pCSII-hCLCN5 by IN-Fusion cloning |
| Com for LV-R1 | cgggcctgtcgggtcGTGGGATGCTCGCAGGCATTCAGgtcccctcggggttg |  |
| Mir142-F1 | ctagaTCCATAAAGTAGGAAACACTACACGATTCCATAAAGTAGGAAACACTACAACCGGGTCCATAAAGTAGGAAACACTACATCACTCCATAAAGTAGGAAACACTACAtctagagtt | Annealed to insert between the XbaI and HpaI sites of pCSII-com-hCLCN5 |
| MIR142-R1 | aactctagaTGTAGTGTTTCCTACTTTATGGAGTGATGTAGTGTTTCCTACTTTATGGACCCGGTTGTAGTGTTTCCTACTTTATGGAATCGTGTAGTGTTTCCTACTTTATGGAt |  |
| mNPT2A-F3 | (cggcaattgaaccggtGAAGGGATGCTGCTTACTGG | Used as primers to amplify a 1308 bp DNA from mouse genomic DNA, 5’ of the mouse Npt2a promoter |
| mNPT2A-MR | TCTCTGCCAACAGAGCTTCA |  |
| mNPT2a-MF | TGAAGCTCTGTTGGCAGAGA | Used as primers to amplify a 2220 bp DNA from mouse genomic DNA, 3’ of mouse Npt2a promoter |
| mNPT2A-R2 | gtggctcgagaccgGTTGGGCACCCACAATGAGTC |  |
| mCLCN5-F5 | cggcaattgaaccggTACTGGACATCACATGGTACC | Used as primers to amplify a 1943 bp DNA from mouse genomic DNA, 5’ of mSglt2 promoter |
| CLCN5-MR2 | ACGTGTTGCTGAATTCTCTCCA |  |
| CLCN5-MF2 | TGGAGAGAATTCAGCAACACGT | Used as primers to amplify a 706 bp DNA from mouse genomic DNA, 3’ of mSglt2 promoter |
| mCLCN5-R5 | gtggctcgagaccggTCACCAACTGTGCCTCTATTG |  |
| hCLCN5-F5 | cggcaattgaaccggtGTCTGTAACACACACGTGTCCTCT | Used as primers to amplify a 2039 bp DNA from HEK293T genomic DNA, SGLT2 promoter |
| hCLCN5-R5 | gtggctcgagaccggtATTCTCCCCAGGATCTGCCC |  |
| PpibF1 | tcgtctttggactctttggaa | Used as an internal control for gene expression. |
| PpibR1 | agcgctcaccatagatgctc |  |
| hCLCN5-F | TCTCGCCATGGATGTTATGA | For Droplet digital PCR to quantify LV titer. |
| hCLCN5-R | TCTTGCGTGCGTTTTCTATG |  |

**Table S3. Promoter sequences**

| Promoter name | Sequence |
| --- | --- |
| Mouse mNpt2a promoter | tGAAGGGATGCTGCTTACTGGCTTGTTCTCATGACTTGCTCAGCTTGCTTTCTTATAGGACCCAAGACTACCTACCAGCTCAGGGATGGTTCCACCCACAATAGGTGGGGCCCTTTACCATCAATTTTACCAGTTCAGAAAATGCCCTTCAGACTTGCATACAACCCAGTCTTATATGTAAAGCTGACATGAAGCTATCTAGTACAAAATTTTCTCATTTATTTTATTTTATTTTATTTTATATGTATAAGTAATTTGCCTATCTATCAAGTGTGTGTGAGATGTCTGGGGAGGCCAGAAGAGGGCATTAGATCCCTTGGAACTGGAGTTACAGAAGGTTGTGAACTGAACCCAGGTCCTCTGGGAAAGCAGCCTAGTGCTCTTAGTCCCTGAGCCATCTCTTCAGTCTCTAAATCCATTCTACTTACTATTCCATAATTGCTAATCCTTTGCTTCCGATCCTCATGACTGCTGGAACTGCTCTCCAGAGCAGGGTAATTGTCAGAGGGAAATGGGTATCTGGACTGGCAGTTATAAACCAATTTGAAACTGATCTTAGTGGCCCACACCTTTAATCCCAACACTTGGGAAGCAGGGTTTGAGGCCAGTCTGCTGTATGTAGTGAGTCTCAGGACAGCCAGAACTACATAGTGAGACACTGTCTTAAAAAAAAAAAAAGCAAGCCAGGAGTGGTGGTGCACACCTTTAATCCCAGCACTCGGGAGGCAGAGGCAGGTGGATTTCTGAGTTCGAGGCCAGCCTGGTCTACAAAGTGAGTTTCAGGACAGCCAGGGCTACACAGAGAAACCCTGTCTCAGGTGGGGTGGGGGGTGGGGGAGATTTGGTCAAGCAAATTTATGGTCACTCTTCCTAAAATGTGAGGGTGCAGCACAGGGTCAAATCGCTTTCGTAGCACTGTGCAGTGCCTTGGGTTCCATCTTCAGCATCAAAAAGCCAATTCCGGTTTTACTTCCCATCCTTTCCCCTTCCTTTTGAGCATTCTTTGGCCTAAATTATTGCTTTAGGTAGAGCTGGAATCTGACCAAGCTGGGTCTGTTCTGCACTCCTCGCCACTGCACAGAGAGCCCTCGGAGAGTCACTATGCCCAGACCGTGAAGGACTGATCAGTTAACCTTGCTTAACTTGAAGCAGGAATGTGCAGAGGGGCCTGTCTTCTGTGGTAGAGAACAGTTAGATAAATGTTACCCTGAAGACACTGAGTGGATCTGCAGTTCCAGGGACCCTGAGTCATCAGAGGACAGCTGAGGAGTCTTGAAGCTCTGTTGGCAGAGAACCTCAAGGTGACCCCAGAGCCTGGTCTCTCAACCCTATCCCTGGCCCATCACCCCTGCTCTTGTTGTGGTTTCCTCTTCTGCTTCCTCAGACATCCCAACCTCAGCCCAGCCTGCTTTGCTCCCCCTGAGTTAAAGGAATTCATCCTCTTCCTTCTCTTGAGACACCCTGCCCTGCTGTAGAACACTCTAGTGTGGTGGTTCTCAACCTATGGGTTGTCTGAGGGTTCCCATTTCACAGGGGTCACCTAGGCCCATCAGAAAACACAGGCACTCCCATTAGGATTCACAACCATAGTAAAATTATAGTTATGAAGGAGCCATGAAAATAATTTTATGGTTGGAGTCACGACGATGGGAGGAAATATATTAAAGGCTTGCAGCGTTAGGAAGGCTTGGAACCATTGCTCTAGGGTACCTGCATTTGTCATGACACTTGGTTGCCATTTCTACTCGCTTAGAGATGACATCATTTGCAGCCGTCTCCTGGCCAGACTTTGAACATCCTGAGGGGCAGTGGCTGTGTGTGGTTTTACTTAGGACTGTACCCCAGCATCTAACTCAGAACTTAGGATCTACTATGCACTCATGAATCACTTGTTGGGTTTGGGGATGTGGCTCCCGTGAACTCTTTCCCACAGGACTTACTGCATATCATGCACTTCCAGATCCCAACTGTTCCTCTTCCTCCTTCCCAATGCCTCTGGGAAGCTTCCTGTCCCCTGTTTCTCCTGAGCCCACCTTAGTTCAGCCAGGCCACCTCTTCCCCGGGTCTCTCCTCCGCATCTCAGCTGCTGCCCCTGCTTCCAGACACTCCTTCTGACAGATCCCATAGCAACACTTTAAGAAGGATGCTAAACCACAGAGCCATCTCTTCTTGGTCTGGGCAGTGACTCTGTGATCTCTTAATCTCACATCGTGATCCATTGTTCAGCCCTGCAGACCCTCCACGGTTCAAAGATGCAGCCTCACTGGGATTCTCCCCTGGGCTCCCCCTTCTGTTGAAGTGTACCCCTTAAGACATACACAGCCTCACAGACATCCAGATGCATGGCAAGGTCACCAGATGTCTGGGTCACTCTCTGGGGAGTCCTGTCCATTCATAAGCCAAGAATGGACAGGAGACACAAAATTGCTTTTGAGTCCCTAAGAACAAGGTACCAGAGGGGTGTCGGAGTCTCTGTCCATGACCAGGATGAGCCTGTACCCTGTGGGTTGTGATTTGTGCTGGTGTAGTGAGAGAGGACAGTCTGACCATAGGCTCCCCCTGCAGAGGTGAAGATTAGCAATTAACTGGAAGGAATCTTGGGGGTAAAGTTAATTTAATGGGGAAAAGAGGGAAGGCAGCTAAAGTTCTTCTAGTTCATTAAGGACTTTGCCCTTGACCCGAGGGTATAAAGAAGAGGGTCTTAGTTCCTCTTTAGACTTCCCCAAGCAGCCGGGCTGGAGCTGAGCCACAGTCAAGGTGAGCCCAGGGCCCCTGTGTCCCAACCCTACCCCTATCCCTTCACCCTAGCTCTTTCTGGGTTTCCTCTTCCTGCTACACATGACATCCCAACACCAGCCCAGCCTGCTTTGCTCCCCCTGAGCTAAAATAATCCCTGCTCTTCCTTCTCTCGAGAGCACCTGTCCTGTCCTGTCCTGCCCTGTCCTGCCCTGCCCTGCCCTGCCCTACCCTGCCCTGCCCTGCCCTGCTGTGAACACTCTAGGCATAGTGGTACCTGAATTCGTCACAATACCTGGTTGCTATCTCTATTCACTGAGACATTATACATTTGCAGCTGTCTCCCCACTAGACTTTGAGAGCAGTGGCTGTATGTGTCTTTAATTGCTACTGTACTTGAGTGTCTAACTCAGAACTCAGCAAACACTACGCATTCAGGAAATACTTCCTGGGACTGGGGATGGGCCTCAACACTGGAGTGCCTGCCTCCTATGATGAGGCCCTAAGTTCCATTTCCAGCAAAACAAATCAGCTACAAAACCCTACTGGGTGGATGAATGGCTGCTTAGGTCTGTGACAAGCACCTGGGTGTCCATCTCTAGATGTGTCTGAGGATGAAAAGAATCACAGACCCTAAGTTGGATATCATCTATGTGGGTATGAGTGTATGAATGTCCCAGACACAGCAACTGTCAGATTTGTCCCAACCTTTTGATATGCAGGACTCATTGTGGGTGCCCAAC |
| Mouse Sglt2 promoter | TACTGGACATCACATGGTACCAAACAAAAGACCCAGTACCAGGAATGAGTTACCATCTTATGAAGTCATTAACCATAGAGAGCTAATGGTAGCCTCAATCATTACAAAAGCTGTTGCCAAGGTTACTGGGTGCTCTCATACCCTGATGGTAAGACCCTATGGCTGAAGATGGAACTTATTGATATCCTTGAACATTGAGAAATTGAGCTGTGTGACTAGAAGCTTCACCCCATCGACGATGGTCACAGTGCTGCAAAGTGCTATGTTCAGGAGGAAGGTATCCAGCCGCCTCCCTCAGCCACACTCTGAGACTCACACCAGAGACCTGCTCAAAGGGCATGCCCACTGGCCCACAAATATTATGGGAGCAACTGACCACTTTTTGGGGGTGTATTTAAGGTCCACTTCATGAAATGGGACCCATCCCTGACACTGCTAAAATGCCTGAGAACCTGAAACTAGATAGAGCAAGGGCTCTAGGGGAAAGCGCACTACAGTTATTCTAAGGGCACAGGGTTATGACGCCTAATGACATATCGCTGGCTACATCCCTTGGCCAGCACATCACTGAAACCTCACCAGAGGAGCTTCTTGGAGTAGAAGGTGATTAACAGGACTGTCCGTGACTGGACGACTTGCAGAGAGTGAGAGACTTGGGAGCACTCAGACTTCAATGGGATGCTTGTATCTCACCCCTCTGTAAAGATGCAGGTTCTATACAGGAGGTGCAAAGATTGTAAGAGTCAGAGTTGGAGGTTGTCTTCAGGAAAGCAGAGTTTTGCAGACACAACAAAGCTGATGAAAATCTGAACTCACAGACAGTGATAGCATGCACAAGACAGATATCTGAACTCACAGACCGTGATAACATGCACAAGACCTGCACAAGTTCGAACCAAATAAAATCTGAGCATGGAAAATGAGATGTGGGCACAAAGTCCCACTCCTAAGTAAGAAACTACTTGCAGTTGACAGCTACTAGGAGAGAGAAATGGGTTTTTTTCAATGGAGAGACACTGGGTGTATCAACTGCACCCCAAGGCAGGCCACACGCTCAGGAAGAGTTGGCCAACACAAAACACACTCCGTGTTTGGTTTTCTGTTTGCTTGGGTTTGTTTTTGTGCTTTTATTCTTCCTTCCTTTCTTTCTTTGTTTCTTTGTTTCTCTCTCTCTTTCTCTCTCTTTTTCTCTCCCTCCTCCTCCTCCTCTTCTTTCTTCTTTTTCTTCTTCTTCTTCTTCTTCTTCTTCTTCTTCTTCTTCTTCTTCTTCTTCTTCTTCTTCTTCTTCTTCTTCTTCTTCTTCTTCTGATCAGAGAGGGGAGGGGATCTGGGAAAAGTTGGGGGAGAGGAAAGAATTTGCCCAAAATATATTGCATAAAAATTTTTTAAAAATAAATTTAAAACAATTTTTAGGATAGAGCAAAGAGAAAGTAGAAAATATTTGGGTTGGGAAGGGCAGGAGAATGAGGGAAATGTGATTTTTTCTCCCTAGGTTTTGTATGGCAGAAGTCAGGGCATGGCATGCGTGACAGTCAGGCTGAGGAACATGTATGTCCTGCTGACTGTCAGGGGGTGCTATGGAGGACTTGTGCGGAGGACACTGTCAGAGTTGGATTTGGACCTTCCTAATCAAAGTTAGAGGGTGTATTTTCAGAGAACGCAGGAAGGAACTTTGCTTGGAACACTGGGTATAGGATGGATCCTAAACCCAGGAAGGAGTGCTCTTGACTTCCAAATGGTCCAGCGCCCCAGGACCAGCCTTCGGCCTTGATAGATCCTGATTCAGCTAAATAAAGCTGGAGAAGGAGGCTGAGACCTGGGGGACTTGTCGGCTCAGTGCTCCTGAGGTAACCATTAATCCTTCCCCCAGGGGAATCCAGGGACTAGCCCCTTGAGGGACAGATGCTGGAGAGAATTCAGCAACACGTAGAGGCAGGCTCTGAACTTGGGGAGCAGAAGGTCCTGATTGATAATCCTGCTGACATTCTGGTTATCGCTGCCTATTTCCTGCTGGTCATTGGTGTTGGCTTGTGGGTGAGACATTGAGGGGGGTTGGATAGGGAAATGCTTCTGGGGCTTGAGGGTAAAGATTTAGGGAGACCTCAGAGAGGAGTGGGAGAAAAGGGTGCTTGGATATAATGAGGGAGAAACCTAGATTTAGTAGGCAAGCCAATTTTAATTCTTTGTCTTCGTACCTTCTGGATTGTGCAAAAGAGACTGGGGGTATCAATAGGTTTTTTTTTTTAATTCAAGTGTTCTAACAAGTGCTCTAAGAGATGTATCAGTTCCCACGTCTGTATTATGGCTGAGCAGCAGCCTATATTTAAGGTCACCAGGCAAGTTAGGCTGAATCTAGGCATATCTAGGATCCAGTAGTTGCGCTAGGATTAGGGCCTGGGTTGTTCTGAGTGTCGGGGAAGGTTGGGGGTAAGGAGGTGCAGTCTGGGGAGTCCAGGGCTGGTTAATCTTCAGCCTGAAACAAGGCTGAGGAATGTGTTGAGGAAGCTAAGGAAGTCCAAAGATGTGCCCCAATCCCAGTTTCCCCCCACTTCTGTTTCCCAGTCTATGTTCAGAACCAATAGAGGCACAGTTGGTG |
| Human SGLT2 promoter | GTCTGTAACACACACGTGTCCTCTGACACTTGGCACTGGATTTCTCTCAGGCACTTTAAACTCATCACCCTCTGAAGCAGACACTGTGGGTGCCCCACCCATATACCTGCATGGCCCACCTTGGAAGTCATATACTGTGACAGTTTCCAGATACCCTGACTTGCAGCTCCTTCTGGAGTTTCTTGTCTGAGGCTGTTCTTGGGAGGAGGGAGTGTGCTCCGTGTTCTCAGCAGGCCTGAAGTGTGTGGACATTTCTGCAGCTTTTAGCCAGTGACAGACACAAATCAGGGAATAGATACCCCAACCACCTGGCCCCTCAAGGAACATTGCTATGGGGTGTTCTTCACAGTGTCTCAGAGTGTCCTTTTTGGGATGAGGCTCCAACTGCTCTTTGTGGTAACCTGACAAATGACACACCCTTACGCCAGGCGTGGTGGCTCACACCTGTAATCCCAGCACTTCGGGAGGCTGAGGCAGTTTGGCTCAGGATTTTGAGACCAGCCTGGGCAACACAGTGAAACCCCATCTGTATAAAAAATACAAAAATTAGCCAAGTGTTGTGGCGTGCCCGTAGTTCCAGCTATTTGGGAAGCTGAGGTGGGAGGATCCCTTGAGCCCAGGAGGTCGAGGCTGCAGTGAGCTGTGATCACACCACTGTACTCCAGCTTTGGTGACACAGCAAGACCCTGTCTCAAAACAAAGAAACACATGCTTTGTTGGTTTTTCTCCTTGTTTCATTTCTCTTCCCTACACCATCACAGTCCATACTGGAATCACCTCCTCAGGAAAGTCGTTGCACACAAATTCTCATCTCAGGGTCTCCTTTTAGAGGAATACAAGCTAAGGAACTCCTTCATCCAAACTCTGTCATATTACACTCAAAGCATTTTTCTCTGTTCCCCATCTCCGGGATGACCATCGCTGCCATAATCAGGCAGGACTTTCCTTCGACTGTCTCCTGACATCCAAGTCTGGTCAATTCTTCTAGAAAGGTAGACATACAGATATTAACCAGCCTCTCCCTTCTTGAATCCAGTCCTGTGCTGAGAAATGAGACTCCAGGGATGAATCAGATCAGTCCTGCTCTTTTTAATTTTTATTTATTTATTTTTTAAGAGATGAGGTCTCACTCCGCTGCCCAGGCTGGTTTCAAACTCCTGAGCTCAAGTGAGCCTCCTGCTGCAGCCTCCCAAAGTGCTAGGATTACAGGCATGAGCCCCCACACCCAGCCAGTCCTACTCTTGAGGAGACACTAGTTTGTTGGGAAGACAGACCTGGACCCAGGCAAGGGCCACACAGTGTGCTCAGAACTACATCAGAAATGTCAGAGAAGTCTTTCTTGAAGAGGCGACACAGGCTGGGTACTGAAAGCTGAGTAGGAGTTGGCCATTTGGGAGACAGGGAAGTGCTCTAAACAGAAGACACAGTTTGTGCAAGGATTTGGTGGGGATAAAATATCTGGTCAAAAAAAGCAAAAAGCAAAAATCTGGGCTGGGTAGGTTAAAGGAGTGGGAAAGGATTTCTGATTTCCTCTAGATTTGGTTTGGAGAAGCAGGGGGAAGGATGAGCGGGAATTGGGGCATGACCAGGATTGGAAATCAGGCTGAAGAGCTTGTACTAAGAGCTATGGAGGGTTCCTGAGGAGGGCGAGTGACCCTGTCAGACTTGGATTTGAAAATGATTCCTCTGGATTAGTTAAATCCAGGGGTGCTAGCTTAGCTAAGGAAGCGATGCATTTTTAGGGAGTAAAAGAGTGATTTTGAGCCTGGAGCACAGGGGAGAGGGCGGATGCTAAGGCCCAGGAAAGAGTGCTCTTGAACTTGGAAGGGCCCAGCTCCCCAAGACCAGCCTTCAGCCTTGATATGACCTGATTCAGCTAAACAAAGCTGGGGAGCGGGAATGAGACCTGGGGGACTTGTCGGCTCAGTGCCCCTGAGGTAACCATTAATCCTTCCCCTGGGGGAATCCAGGGGCTGGTTCCTGGATGGGGCAGATCCTGGGGAGAAT |
|  |  |
